# The Impact of a Submaximal Level of Exercise on Balance Performance in Older Persons

**DOI:** 10.1155/2014/986252

**Published:** 2014-10-14

**Authors:** Hani Asilah Alias, Maria Justine

**Affiliations:** ^1^Department of Physiotherapy, Faculty of Health Sciences, Universiti Teknologi MARA (UiTM), Puncak Alam Campus, 42300 Puncak Alam, Selangor, Malaysia; ^2^Community of Research (CoRe) of Humanities and Quality of Life, UiTM, 40450 Shah Alam, Selangor, Malaysia

## Abstract

*Objective*. The purpose of this study was to determine the impact of a submaximal level of exercise on balance performance under a variety of conditions. *Material and Method*. Thirteen community-dwelling older persons with intact foot sensation (age = 66.69 ± 8.17 years, BMI = 24.65 ± 4.08 kg/m^2^, female, *n* = 6) volunteered to participate. Subjects' balance performances were measured using the Modified Clinical Test of Sensory Integration of Balance (mCTSIB) at baseline and after test, under four conditions of stance: (1) eyes-opened firm-surface (EOF), (2) eyes-closed firm-surface (ECF), (3) eyes-opened soft-surface (EOS), and (4) eyes-closed soft-surface (ECS). The 6-minute walk test (6MWT) protocol was used to induce the submaximal level of exercise. Data was analyzed using the Wilcoxon Signed-Rank Test. *Results*. Balance changes during EOF (*z* = 0.00, *P* = 1.00) and ECF (*z* = −1.342, *P* = 0.180) were not significant. However, balance changes during EOS (*z* = −2.314, *P* = 0.021) and ECS (*z* = −3.089, *P* = 0.02) were significantly dropped after the 6MWT. *Conclusion*. A submaximal level of exercise may influence sensory integration that in turn affects balance performance, particularly on an unstable surface. Rehabilitation should focus on designing intervention that may improve sensory integration among older individuals with balance deterioration in order to encourage functional activities.

## 1. Introduction

The worldwide increase in the aging population has raised concern among policy makers, scientists, and health care providers. This is because the increasing number of this vulnerable population may also reflect the needs to provide a better environment for them to live safely and independently. Older persons are vulnerable to functional decline due to its normal physiological changes that affect the body systems such as the musculoskeletal, cardiovascular, and endocrine systems.

One of the consequences of the normal physiological changes is the feeling of fatigue, especially while or after performing activities of daily living. Schultz-Larsen and Avlund [1] revealed that 49% of men and 53% of women had conveyed tiredness following one or more daily physical activity. In addition, among community-dwelling individuals population, 27% aged 50–64 years and 37% aged ≥65 years had reported exhaustion within the past month [2]. A previous research by Hardy and Studenski [3] showed that 70% of 495 community-dwelling older persons aged 65 years and above had reported fatigue and 43% of them reported feeling tired most of the time. The increasing number of fatigue cases reported with increasing age showed that fatigue has become so prevalent among older persons.

Fatigue is crucial and is a medical concern nowadays because it has been associated with functional decline and mortality [4, 5]. The evidence is strongly supported by Vestergaard et al. [6] which found that fatigued subjects aged 65 years and above had a significantly greater disability in physical function, mobility, and instrumental activities of daily living compared with nonfatigued subjects. However, the effect of fatigue is not limited to health problems only. Fatigue also can affect work performance, family life, and social relationships negatively [7]. Fatigue has also been shown to appear after performing exercise or physical activity and results in worsening motor performance [8]. Interestingly, a recent finding showed that fatigue is associated with falls among community-dwelling older persons [9]. Logically, fall-prone older persons may have poor control in postural balance [10]. As such, it is important to determine the impact of fatigue or certain level of exercise performance on balance performance in older persons.

Previous studies related to fatigue have evaluated the effects of peripheral fatigue by means of isometric agonists and/or antagonists lower limb muscle contraction [11–13], while six-minute walk has been used to test the effects of fatigue on gait dynamic stability [14]. Another study evaluated the effects of fatigue induction using 2 km treadmill walking on balance performance [15]. However, the variability in the fatigue induction is arguable; for instance, localized muscle fatigue may not represent the functional activities of daily living that are important for older persons. Besides, the peripheral fatigue induction procedures are more focused on the usage of machine which older persons may not be familiar with. In addition, walking on a treadmill and in a static environment may produce compensation in maintaining the balance difficulty during the locomotion [16]. Furthermore, to date, there has been limited agreement on fatigue resulting from the short duration of submaximal exercise and its effect on balance performance. This supports the importance of studying the effects of balance performance following a more generalized fatigue induction.

Assessment of balance among older persons is vital. Static balance has been shown to be correlated positively with functional fitness and daily physical activities [17]. Both of these components are important in maintaining the independence of older persons in regulating instrumental activities of daily living. As such, this pilot study aimed to determine the impact of a submaximal level of exercise on balance performance under a variety of conditions. The six-minute duration of self-preferred walking speed was used in this study to represent the submaximal level of exercise [18]. We hypothesized that balance performance may not be significantly affected following the performance of the submaximal level of exercise. This study was conducted to provide insights regarding the effect of fatigue due to a submaximal level of exercise on static balance.

## 2. Material and Method

### 2.1. Study Design and Participants

Twenty-three community-dwelling elderly aged 60 years and above agreed to participate in this one-group pretest-posttest study. From this subtotal, 10 were not eligible due to the exclusion criteria. Therefore, total participation enrolled in this study was thirteen elderly, which consisted of 6 males and 7 females. The subjects' inclusion criteria were as follows: are aged 60 years and above, are able to walk independently without the use of walking aids, score ≥24 in the Mini Mental State Examination (MMSE) [19], currently are not involved in regular exercise programs of more than 30 min/day for 3 times/week, have no self-reported diagnosis of type II diabetes or sensory impairment, and are independent in activities of daily living. The exclusion criteria were as follows: inability to understand the study procedure, self-reported having disabling pathologies (e.g., osteoarthritis and chronic inflammatory rheumatic diseases of lower body) and other chronic diseases, and abnormal blood pressure. The study was conducted in the community hall, Kampung Tok Muda Kapar, Selangor, Malaysia. The protocol for this study was approved by the Institutional Research Ethics Committee [600-FSK (PT.5/2)].

### 2.2. Measurements

#### 2.2.1. Anthropometric Data

Subject's age (year), gender (male/female), body weight (kg), height (m), and body mass index (BMI) (kg/m^2^) were recorded.

#### 2.2.2. Balance Performance

Subject's balance performance was measured using the Modified Clinical Test of Sensory Integration of Balance (mCTSIB) [20] both during pre- and posttest procedures. The mCTSIB shows excellent test-retest among community-dwelling elderly (*r* = 0.75) [21] and interrater (68%–100%) [20], while the criterion validity with fallers is 63% and with nonfallers is 77% [22]. The mCTSIB measures patient's standing balance which influences visual, vestibular, and somatosensory input [23]. The test consists of four conditions of quiet stance: (1) stance on a firm surface with eyes open (EOF); (2) stance on a firm surface with eyes closed (ECF); (3) stance on a soft surface (10 cm thick foam) with eyes open (EOS); and (4) stance on a soft surface with eyes closed (ECS) [20].

During the test, subject stood with feet together while maintaining the back in a straight alignment. The hands were placed on the hips or around the waist. The standing quality was observed for a maximum of 30 seconds in three trials of quiet standing without rest in between each trial and four conditions of the balance test. The test was stopped if the subject performed any of the following movements: (a) opened eyes in an eye-closed condition, (b) raised arms from the sides, or (c) lost balance and required manual assistance to prevent a fall. The time (seconds) in which the subject was able to stand quietly was recorded as the score for the test. The average scores of the three trials were calculated and documented for analysis. The scores for the balance performance were recorded prior to performing the six-minute walking and following the completion of the procedure.

#### 2.2.3. Level of Fatigue

The subject's fatigue level was evaluated using the Borg Category-Ratio-10 scale (Borg CR-10) [24]. Borg CR-10 consists of a sequence number ranging from 0 to 10 points. A “0” indicates no fatigue at all while “10” is an extremely strong level of fatigue. The Borg CR-10 scale is able to predict exercise capacity during self-paced procedures clamped by RPE [25]. Subjects were instructed to rate their level of fatigue during the beginning of the six-minute walking and immediately after the walking procedure ended.

### 2.3. Procedure for the Six-Minute Walk Test (6MWT)

We used the protocol for the 6-minute walk test (6MWT) to induce a generalized fatigue. The procedure followed the standard requirements as recommended by the American Thoracic Society (ATS) [18]. The subject walked along a rectangular walking course in length of 30 meters. In order to provide a visual sign to the participant, a bright color tape was used to mark the starting line. A cone was placed at each turnaround point to guide the subject to walk through the right walking path.

To begin the 6-minute overground walking, the subject was asked to stand behind the marked starting line. Then, he/she started to walk with his/her self-paced walking speed once the assessor gave a signal of “go.” The assessor monitored and recorded the subject's heart rate for each minute throughout the 6 minutes of walking and informed the subject of his/her remaining time to walk. At the same time, another assessor recorded the number of laps on the worksheet once the subject finished a 30-meter walking course. The subject was not allowed to speak unnecessarily during the procedure.

Subject had to stop walking once the 6-minute walking duration is over. A command of “stop” was given by the assessor to inform the subject that he/she has to stop walking. The distance of an unfinished full cycle of the walk was measured using a measuring tape. The score for the total distance walked throughout the 6 minutes was recorded but was not analyzed for the purpose of discussion, as the intention of this study was to induce fatigue following a submaximal level of exercise.

### 2.4. Statistical Analysis

The balance performance as measured by the mCTSIB was tested at baseline and following the completion of the 6MWT. The mean (m) and standard deviation (SD) for related variables were reported. The Wilcoxon Signed-Rank Test was used to analyze the balance differences within the group. The level of significance was set *P* < 0.05.

## 3. Results

Thirteen community-dwelling elderly subjects participated in this study and their characteristics were reported in Table 1.

The Wilcoxon Signed-Rank Test revealed no significant changes in balance performance following the submaximal level of exercise during EOF (*z* = 0.00 and *P* = 1.00) and ECF (*z* = −1.342 and *P* = 0.180). However, the balance performance during EOS (*z* = −2.314 and *P* = 0.021) and ECS (*z* = −3.089 and *P* = 0.02) were significantly dropped following the submaximal level of exercise. The fatigue level also increased significantly (*z* = −2.46 and *P* = 0.014) after the 6-minute walking.  Table 2 presents the mean and SD for the balance scores and fatigue level during the pretest and posttest.

## 4. Discussion

The objective of this pilot study was to compare between balance performance before and after six-minute walking in older persons. We found no significant changes in balance performance while standing on a firm surface measured during baseline and after exercise. However, a significant decline in balance performance was detected for both standing on soft foam with eyes closed and with eyes opened from pretest to posttest. Another study that has used a different type of outcome measure showed meaningful decrease in postural balance after the implementation of the fatigue protocol of ankle plantar flexor and knee extensor muscles among thirty active elderly women [11]. The current study indicates that the proprioceptive system, which is one of the control mechanisms of balance, may be affected by fatigue following the completion of a certain level of exercise.

Other than that, the analysis on the level of fatigue by means of the Borg Category-Ratio-10 Scale did show a significant change. However, the magnitude of change can be argued because both pre- and postscore on the mean were less than 1. Therefore, we conclude that the Borg Category-Ratio-10 Scale was not sensitive enough to detect the small change in the level of fatigue due to its reliance on subjects' individual perception. In addition, the six-minute walk may not be strenuous enough to cause fatigue, especially amounting to elderly individuals who live in the community. It is recommended that future study should implement a more objective measure such as measuring blood lactate.

### 4.1. Balance on Firm Surface

Within our extensive reading, limited studies had analyzed the balance performance solely on the duration of sustaining stable quiet standing on an even surface. Contrary to our expectations, the effects of balance on an even surface with eyes opened after moderate intensity exercise were not significantly changed in this study. Our finding was inconsistent with previous study which suggested that the moderate intensity, self-paced exercise induces statistically significant changes to the amplitude of medial-lateral (ML) displacement and variation of the ML position of the COP with eyes opened [26]. Nevertheless, the activity protocol in the previous study incorporated walking and more functional activities in a circuit method within 14 minutes. Indeed, the duration of inducing fatigue was longer compared to the current study. It seems possible that the six-minute duration of walking might be insufficient to provide a significant impact on balance performance. Similarly, a recent study has found that center of pressure path (COP_path_) was significantly increased after submaximal 2 km and particularly during maximal exercise during standing while eyes were closed [15]. The possible explanation of the inconsistency of the current study and previous finding could be that older adults relied more on vision to maintain postural stability. Excessive reliance on visual input may be a natural compensatory strategy to cope with poor balance among older persons [27].

### 4.2. Balance on Soft/Uneven Surface

The most important finding in this study was the significant decline in the balance performance on a soft surface following the fatigue protocol. As aging occurs, balance function starts to deteriorate and may cause problems for older adults. Older adults faced more difficulty than younger adults in maintaining steadiness, under conditions wherein sensory information for postural control is severely reduced [28]. Postural balance is controlled by sensory information, central processing, and neuromuscular responses [29]. The sensory system provides information to the central nervous system, which in turn sends nerve impulses to the muscles to coordinate and control the body segments [30]. However, prolonged exercise may induce neuromuscular fatigue through various mechanisms, that is, alterations in the activation of the primary motor cortex, reduction of motor unit discharge rates, alterations of excitation-contraction coupling, and slowing of the contractile apparatus [27, 28]. The inability of the stabilizing musculature to produce or sustain the required muscle tone under fatigued conditions may lead to decreased functionality of the proprioceptive muscle receptor system [31] and further explained the reasons for the decline in the balance performance as shown in this current study. Other studies explained that the accumulation of metabolites may lead to altered muscle spindle function [30, 32] as well as altered central processing of proprioception via groups III and IV afferents [31]. Therefore, altered somatosensory input due to fatigue could result in deficits in neuromuscular and postural control.

### 4.3. Clinical Implication

This finding supports the literature suggesting that postural control deficits observed after fatigue are largely due to a deficit in proprioception and suggests that fatigued-related balance impairments leading to falls/injuries could be more frequent in situations where proprioceptive information is reduced (e.g., diabetic or other patients with peripheral neuropathy). It is further suggested that postural control on a compliant surface would be impaired to a greater extent after fatigue compared to a firm surface because standing on a compliant surface requires more proprioceptive information and greater control of the neuromuscular system.

Based on the current understanding, we suggest that any exercise prescription or intervention for older persons should be recommended according to their level of functional capacity. Therefore, it is important to assess the ability of the older persons prior to any prescribed interventions. Furthermore, any interventional strategies for older persons aiming at improving balance should be included with a variety of challenges or different conditions as older persons need to adapt themselves to various walking challenges especially inside or outside their homes.

### 4.4. Strength of the Study

The major strength of this study is that the exercise protocol utilized was similar to physical activity during performing daily tasks. The submaximal level of exercise was adopted from the six-minute walking test [18]. Walking capacity is central to the performance of many activities of daily living [33]. Other than that, the assessors who measured the outcome variables were blinded to previous assessment of the outcome.

### 4.5. Limitation of the Study

Several limitations to this pilot study need to be acknowledged. The present study was limited by the amount of subjects recruited, which was relatively small. Therefore, a nonparametric test, namely, Wilcoxon Signed-Rank Test, has to be used in order to analyze the impact on balance following submaximal level of exercise. Another limitation that should be considered was that the lack of comparison group resulted in exposition to threats of internal validity occurring in this study. Moreover, the six-minute time interval between pre- and postmeasurements seemed short, and self-preferred walking speed performed was not reliable enough to induce fatigue among elderly individuals who live in the community. In addition, balance performance was assessed via observation of stability during quiet standing within 30 seconds of trial. More objective balance assessment components, for example, center of pressure and medial-lateral and anterior-posterior sway, were not investigated. Furthermore, this study also failed to examine the age-related changes of the musculoskeletal system (i.e., lower limb muscle strength, range of motion, and orthopedic deformities) that might contribute to the changes of balance performance. Finally, further relationship of the balance performance and level of fatigue may need to be thoroughly assessed and discussed. As such, further study is required with improvement in the methodology of the study such as increasing the sample size, blinding of assessors, including assessment of subjects' functional capacity, and selection of a homogeneous population.

## 5. Conclusion

A submaximal level of exercise seemed to influence sensory integration that in turn affects balance performance, particularly on an unstable surface. Therefore, this study would suggest that rehabilitation should focus on designing intervention that may hold potential for improving sensory integration among older individuals with balance deterioration in order to encourage functional activities. Besides, older persons may require extra caution during exercising or performing daily physical activity like having a short rest in between to avoid the occurrence of fall although the risk remains unknown. It is recommended that further research in determining whether the sensory integration changes would be sufficient to increase fall risk must be undertaken. More broadly, research is also needed to evaluate the effect of fatiguing activity on other balance control mechanisms and its association with risk of falls.

## Figures and Tables

**Table 1:** Demographics of thirteen community-dwelling elderly.

Characteristics (*N* = 13)	Value
Age, year, mean (±SD) (range)	66.69 (±8.17); (60–84)
Female, *n* (%)	6 (46)
Male, *n* (%)	7 (54)
Height, m, mean (±SD) (range)	1.53 (±0.07); (1.43–1.68)
Weight, kg, mean (±SD) (range)	59.41 (±7.10); (39.3–73.0)
BMI, kg/m^2^ mean (±SD) (range)	24.65 (±4.08); (17.0–31.0)

**Table 2:** The mean equilibrium time (second) of balance performance during four conditions of stance and fatigue level for pre- and posttest six-minute walking.

Variables	*n*	Pretest		Posttest		*z*	*P* value
		Mean	SD	Mean	SD		
Balance performances							
Eyes opened with firm surface(s)	13	30.00	0.00	30.00	0.00	0.00	1.000
Eyes closed with firm surface(s)	13	30.00	0.00	29.23	1.92	−1.342	0.180
Eyes opened with soft surface(s)	13	25.92	7.96	19.46	8.17	−2.314	0.021∗
Eyes closed with soft surface(s)	13	6.62	5.40	3.15	2.19	−3.089	0.020∗
Fatigue level							
Borg Category-Ratio-10 Scale	13	0.23	0.44	0.92	0.76	−2.46	0.014∗

^*^Significant difference at *P* value <0.05.
